# Protective Factors for Early Childhood Caries in 3-Year-Old Children in Poland

**DOI:** 10.3389/fped.2021.583660

**Published:** 2021-03-15

**Authors:** Dorota Olczak-Kowalczyk, Dariusz Gozdowski, Anna Turska-Szybka

**Affiliations:** ^1^Department of Paediatric Dentistry, Medical University of Warsaw, Warsaw, Poland; ^2^Department of Experimental Statistics and Bioinformatics, Warsaw University of Life Science, Warsaw, Poland

**Keywords:** early childhood caries, 3-year-old children, oral health behaviors, oral hygiene habits, dietary habits, protective factors

## Abstract

**Background:** Early childhood caries (ECC) remains highly prevalent in preschool children worldwide. Dental caries affects every second 3-year-olds in Poland. The aim of the study was to assess protective factors for ECC in 3-year-olds.

**Methods:** A cross-sectional survey was conducted in 2017 among 3-year-olds attending kindergartens in all 16 provinces of Poland. The questionnaire included socioeconomic factors, and oral health behaviors. Decayed, missing due to caries and filled teeth and surfaces were assessed. Caries indices (dmft and dmfs), percentage of caries-free and severe ECC (S-ECC) were calculated. The Student's *t*-test, Spearman correlation, univariate and multivariate logistic regression (the odds ratios (OR) and adjusted odds ratios (AOR): confounding factors: socioeconomic conditions, oral health behaviors) were performed; *p* < 0.05.

**Results:** A total of 1,638 children were included. ECC was confirmed in 41.1%; S-ECC in 23.4%. The dmft index was 1.85 ± 3.14, dmfs = 2.99 ± 6.56. Spearman correlation [socioeconomic factors, especially mother's education (*r* = −0.148, *p* < 0.001) and family's economic status (*r* = −0.071, *p* = 0.004)], despite were statistically significant, indicate very weak relationships with dmft index. After 12th month of life not giving any beverages before bedtime and at night, but water or unsweetened milk only, healthy snacking (sandwiches, fresh fruit), or water on a daily basis decreases the probability of caries development (for S-ECC: OR = 0.37, AOR = 0.47, AOR2 = 0.37; *p* < 0.01), even if inappropriate dietary behaviors or hygiene practices were simultaneously present. Brushing of a child's teeth twice a day lowered the odds of caries (for S-ECC: OR = 0.78, *p* = 0.031; AOR = 0.81, *p* = 0.081; AOR2 = 0.84, *p* = 0.131), but this effect was less intense when inappropriate dietary behaviors were accounted for. Children of parents with good self-assessment of their teeth were more often caries-free (61.4 vs. 42.9%; *p* = 0.006) and had lower dmft indices (1.67 vs. 2.93; *p* = 0.002).

**Conclusions:** Preventing a child after 12th month of life from getting any beverages before bedtime, water or unsweetened milk only, sandwiches and fresh fruit as snacks, or water given to on a daily basis, tooth brushing twice a day decrease the odds of caries in 3-year-olds. Diet appears to have primary significance in the etiology of ECC, since tooth brushing can only partly attenuate the impact of inappropriate dietary behaviors on dental caries. Children are more often caries-free and have lower severity of caries if their parents' dentition is self-assessed as healthy.

## Introduction

Early childhood caries (ECC)—defined as caries affecting children until the age of 71 months—remains a public health problem in many countries. The consequences of ECC include localized painful sensations, infections, abscesses, hospitalizations and emergency admissions, retarded physical development, malnutrition, gastrointestinal disorders, disturbed sleep and malocclusion (1–3). Dental caries occurring in children in crèches, that means soon after tooth eruption, may be particularly devastating, with dynamic course and high risk of pulpal complications. It is a disease that can rapidly and irreversibly destroy the primary dentition. In several European countries, the incidence of ECC in children aged 3 years does not exceed 20% (2, 4, 5). In others, it is much higher (2, 4, 6–9). In studies conducted in Poland in 2015 ECC was confirmed in 53.5% of 3-year-olds (9).

Implementation of preventive measures in a child when the first primary teeth erupt is a prerequisite for lowering the incidence of EEC. Babies and young children rarely present at the dental offices, and yet, their health is monitored by pediatricians, family doctors and nurses. Pro-health education that they provide for the parents should also include dietary and hygienic standards that would restrict and/or eliminate the impact of causative factors of caries in children, and at the same time enhance the prophylactic ones.

A number of researchers have assessed factors associated with early childhood caries (2, 3, 5–12). A whole range of factors including socioeconomic, behavioral, and psychosocial ones in particular, are identified in the etiology of EEC. The initiation process is directly influenced by predominance of cariogenic microorganisms in dental plaque biofilm that are sustained by prolonged exposure to fermentable carbohydrates. Activities that lead to early colonization of a child's oral cavity with cariogenic bacteria, hygienic neglect and improper diet that involves frequent or prolonged exposure to fermentable carbohydrates are well-known and documented caries risk factors affecting primary teeth. Fluoridation, cleaning of teeth and proper diet are regarded as basic protective measures. Snacks with low cariogenic potential are preferred, thirst should be quenched with still water, sweets should be restricted to 1 day a week, and sugar replaced with substitutes like xylitol. A child's teeth should be regularly brushed by an adult person with fluoridated toothpaste and check-up appointments arranged with a dentist to facilitate topical application of a fluoride varnish.

In prevention of EEC, it is of fundamental importance to educate parents in proper hygienic and dietary attitudes, and to stress the significance of recommended practices in the first period of a child's life relating to its teeth, especially if cariogenic factors are present (1–3, 5–12).

The aim of the study was to assess oral health behaviors relative to prevalence of ECC in children aged 3 years, with particular emphasis on practices widely regarded as beneficial and protective for the health of teeth.

## Materials and Methods

### The Study Population

A cross-sectional survey was conducted in 2017 among three-year-olds attending kindergartens in all 16 provinces of Poland. For each province, districts and counties were randomly selected, and the same process was repeated for towns, villages and finally for kindergartens. The selection was based on a complete list of administrative units and kindergartens; for each object random value was generated using RAND function in Microsoft Excel and the objects with the highest value were selected for the study. At least three kindergartens were selected separately for rural and urban areas to obtain similar percentage of children who lived in these areas (respectively about 50 and 50 children for each province). After the permission of the heads of these facilities had been obtained, the parents of 3-year-old attendees were informed in the scope of the study. They were then asked for the written consent concerning the participation in the study followed by completion of a questionnaire. All the children who were present in the kindergarten on the day of the examination, and who co-operated with the dentist, were included in the study, provided their parents had signed the participation consent and filled in the questionnaire. Children were eligible to participate if they were healthy and aged 3 years. Exclusion criteria were: children with a physical or mental disability and those with developmental defects of teeth.

The size of the sample was calculated with an assumption that error tolerance (margin error based on confidence interval (CI) at 95% confidence level) for binomial variables would not exceed the value of 2.5% for most cases. At such assumption required, the sample size included at least 1,500 subjects for total sample. In the present study it was increased by about 10% for higher results' confidence.

The parents were surveyed, and the children examined in accordance with the WHO guidelines (13).

The participants were divided into two groups: caries-free (dmf = 0) and the controls with dental caries) (dmf > 0). The data were collected using two data gathering tools: the child's oral examination form and the parent's valid questionnaire.

### The Questionnaire

The questionnaire followed the WHO Oral Health Surveys Basic Methods guidelines, which recommends the use of simplified structured questionnaires for the collection of data on oral health and caries risk factors in children (14). The questionnaire included questions concerning three domains: socioeconomic factors such as the place of residence, mother's education, perceived cost of maintaining oral health, the number of children in the family and oral health behaviors, including the child's oral hygiene habits (frequency of tooth brushing, supervision of tooth brushing, the use of fluoridated toothpaste), and feeding practices and dietary habits in the 2nd year of life (provision and type of liquids/solids before going to bed or at night, foodstuffs with sugar) as well as the current habits (the number of meals and episodes of snacking during the day, type of preferred snacks, frequency and variety of foodstuffs, the use of sugar substitutes, restrictions on the consumption of sweets to 1 day a week). Since dairy products have a protective effect on teeth, this factor was included in the analysis as a question on unsweetened milk.

Preliminary analyses showed that some questions included in the survey, such as whether children used fluoride-containing toothpaste or whether parents/caregivers restricted consumption of sugar-containing foodstuffs were not significant and were omitted from the final analysis.

### The Clinical Examination

The clinical examinations were conducted in artificial light (lightweight portable examination light) using plane mouth mirrors and CPI (Community Periodontal Index) probe in accordance with the WHO standard for epidemiological surveys (14). Children were examined in an upright position in a chair with a high backrest. The children were instructed to brush their teeth before the examination. The dentists dried the surface of the teeth with cotton rolls and swabs. The examinations were always conducted before 12 am.

All examiners took steps to protect both the patients and themselves in accordance with the sanitary and epidemiological regulations in force.

Prevalence of ECC (presence of one or more decayed, missing due to caries, or filled tooth surfaces) in any primary tooth in a child and S-ECC [one or more cavitated, missing (due to caries), or filled smooth surfaces in primary maxillary anterior teeth or a decayed, missing, or filled score of greater than, or equal, four (age three)] as well as mean of dmft and dmfts indices were calculated (1). The dental examinations were conducted by 16 teams (from each province of Poland) consisting of two dentists trained in survey methodology, specialists in pediatric dentistry, with many years of experience.

Training and calibration of the examiners was conducted according to the WHO recommendations to ensure validity and reliability, which was additionally verified by another 10% sample randomly selected as control examination (14). The intra-examiner and inter-examiner reproducibility was performed. Each pediatric dentist (examiner) independently examined the same group of 10 patients, and the findings were compared with those of the experienced supervisor. Cohen's Kappa coefficients between the reference researcher and the other researchers was between 0.857 and 1.000 for carious teeth, and should be interpreted as an almost perfect agreement. These calculations were based on individual tooth surfaces for 10 children. This survey was conducted using paper sheets, and then they were collected, stored, processed and analyzed in electronic records. The data (paper sheets and electronic records) are kept by the Ministry of Health as the National Oral Health Surveys. The study meets the requirements of personal health data protection according to regulation of The European Union General Data Protection Regulation (EU GDPR).

### Statistical Analysis

An individual child was treated as an experimental unit for statistical analyses (total sample size equaled *N* = 1,638). The variables on dental caries (dmft, dmft = 0, dmfs ≥ 4), children's oral hygienic practices, dietary history like consumption of sugar-containing foodstuffs in the first 2 years of life and current consumption by a child of >3 snacks a day, frequency of consumption of products containing sugar) and socioeconomic factors were presented as percentages or means and standard deviation. Spearman rank correlation coefficient was used for evaluation of relationships between various factors on the occurrence of caries in children. Univariate and multivariate logistic regression was applied for evaluation of effects of independent variables (behaviors regarded as beneficial for oral health) on the binomial variables characterizing the intensity of caries (dmft = 0 and occurrence of S-ECC). Based on simple and multivariate logistic regression, the odds ratios (OR) and adjusted odds ratios (AOR, AOR1, and AOR2) with confidence intervals were determined (at 95% confidence level) for the relative risk of caries development depending on potentially causal variables (socioeconomic conditions, inappropriate dietary and hygienic behaviors). Three types of adjusted odds ratios were calculated where confounding factors were following: AOR–socioeconomic conditions, AOR1–inappropriate dietary behaviors past and present, and AOR2–inappropriate hygienic practices present.

Statistical testing of differences between means for two groups was determined using the Student's *t*-test. The analyses were performed in the SPSS 24 and Statistica 13 software. Significance level for all the analyses was set at *p* < 0.05.

## Results

A total of 1,638 children aged 3 years were included in the study (representing 92% of the original total), with 859 (52.4%) of girls and 866 (52.9%) of city dwellers. The number of participants per a province ranged from 100 to 115 children. A total of 939 (57.3%) of mothers declared university education, and 163 (10%) primary/vocational schooling. In the majority of cases (970−59.2%), the respondents assessed their economic status as average, by 476 (29.1%) as high and by 192 (11.7%) as low.

ECC was confirmed in 674 (41.1%) of the examined children, S-ECC in 23.4%. The mean value of the dmft index was 1.85 ± 3.14, dmfs = 2.99 ± 6.56. Frequency and severity of caries predominated in boys, when compared with girls, at 43.8%, dmft = 2.04 ± 3.21 and 38.8%, dmft = 1.68 ± 3.06, respectively (*p* = 0.023). Table 1 presents oral health behaviors of the examined children statistically significantly correlated with dental caries.

**Table 1:** Oral health behaviors of children related to dental caries based on Spearman correlation.

**Oral health behaviors**	***N*/%**	**Spearman rank correlation**			
		**dmft = 0**	**dmft**	**dmfs ≥ 4**	
**History of dental care**					
Past dental visits	780/47.6	−0.124*	0.159*	0.165*	
Application of fluoride varnish	176/10.7	−0.070*	0.106*	0.129*	
**Hygienic practices**					
Brushing of a child's teeth twice a day	868/53.0	0.053*	−0.063*	−0.053*	
**Dietary history**					
Presence of sugary foodstuffs in the diet in the first 2 years of life	1420/86.7	−0.079*	0.064*	0.026	
Providing a child before bedtime and at night after 12th month of life:					
• Water or no other beverage	495/30.2	0.075*	−0.091*	−0.091*	
• nursing	189/11.5	−0.047	0.063*	0.084*	
• beverages sweetened with sugar or honey	278/17.0	−0.104*	0.135*	0.126*	
• unsweetened milk in a bottle	676/41.3	0.041	−0.059*	−0.066*	
**Current diet**					
> three snacks a day	30/1.8	−0.034	0.051*	0.064*	
Snacking (sandwiches, fresh fruit)	411/25.1	0.086*	−0.084*	−0.044	
Sweet snacks (buns, dairy products)	1103/67.3	−0.085*	0.086*	0.060*	
**Frequency of consumption:**					
• candies, bars	A	144/8.8	−0.070*	0.070*	0.042
	B	481/29.4			
• sponge fingers, cakes, doughnuts, layer cakes, buns	A	206/12.6	−0.057*	0.052*	0.035
	B	288/17.6			
• fruit juices	A	561/34.2	−0.048	0.063*	0.048
	B	332/20.3			
• sweet fizzy drinks	A	45/2.8	−0.093*	0.105*	0.087*
	B	1445/88.2			
• tea with sugar	A	572/34.9	−0.054*	0.064*	0.032
	B	606/37.0			
• milk or cocoa with sugar	A	478/29.2	−0.046	0.064*	0.078*
	B	650/39.7			
• salted crisps, sticks or crackers	A	48/2.9	−0.064*	0.073*	0.075*
	B	1047/64.0			
• water	A	1257/76.8	0.059*	−0.081*	−0.066*
	B	164/10.0			

*A- several times or daily; B- several times a month, rarely/never*.

B- *statistical significance p < 0.05.

Spearman correlation analysis revealed that (socioeconomic factors, especially mother's education (*r* = −0.148, *p* < 0.001) and family's economic status (*r* = −0.071, *p* = 0.004), despite were statistically significant, indicate very weak relationships with the severity of caries expressed by the value of the dmft index. On the basis of logistic regression analysis it was found that the odds of EEC decreased when teeth were brushed twice daily (OR = 0.81, *p* = 0.033). Provision of water and unsweetened milk decreased the risk of ECC (OR = 0.86, *p* = 0.193 and OR = 0.84, *p* = 0.099, respectively) i S-ECC (OR = 0.76, *p* = 0.056 and OR = 0.73, *p* = 0.008, respectively). The multivariate regression analysis showed that the dental visits during last 6 months were significantly associated with higher odds ratio of ECC occurrence (AOR = 1.83, *p* < 0.001).

Table 2 presents the relationship between ECC and behaviors regarded as beneficial for oral health. Brushing of a child's teeth twice a day vs. sporadic and 1–3 times a week significantly decreases the odds of caries, but this effect is weaker when adjusted for the impact of inappropriate dietary behaviors. Children of parents whose self-assessment score was higher tended to have lower value of the dmft indices (1.67 vs. 2.93, *p* = 0.002), also S-ECC was a rarer occurrence (21.7 vs. 37.5%, *p* = 0.006) and often they were caries-free (61.4 vs. 42.9%, *p* = 0.006). This effect was also significant when adjusted for potential confounders (AOR1 = 0.48, *p* = 0.010 for S-ECC). After 12th month of life a child was not given not any beverages before bedtime and at night; water or unsweetened milk and snacking of sandwiches, fresh fruit, and drinking water on a daily basis will dramatically increase the prospect of maintaining healthy teeth. Mean dmft of 1.23 in children with healthy snacking habits was almost three times lower in comparison with children with unhealthy snacking habits (dmft = 3.56). The importance of dietary factors was not diminished by hygiene behaviors or socioeconomic factors.

**Table 2:** Relationship between ECC and behaviors regarded as beneficial for oral health.

**Protective behaviors related to dentition status**	**% dmft** **=** **0**		**% S-ECC**		**dmft**	
	**yes**^***a***^	**no**	**yes**	**no**	**yes**	**no**
Brushing of a child's teeth twice a day (vs. sporadic and 1–3 times a week)	532/868 (61.3%)	432/770(56.1%)	185/868 (21.3%)	199/770(25.8%)	1.61 ± 2.82	2.12 ± 3.44
OR^*b*^	*1.24 (1.02–1.51)* p = 0.033^*^		*0.78 (0.62–0.98)* p = 0.031^*^		*p* = 0.001^*^	
AOR	*1.19 (0.97–1.45)* *p = 0.096*		*0.81 (0.64–1.03)* *p = 0.081*			
AOR1	*1.17 (0.96–1.43)* *p = 0.126*		*0.84 (0.66–1.06)* *p = 0.131*			
Parental assessment of the condition of their teeth (poor vs. good)	752/1224 (61.4%)	24/56(42.9%)	262/1224 (21.7%)	21/56(37.5%)	1.67 ± 2.91	2.93 ± 3.81
OR	2.21 (1.24–3.65) *p =* 0.006*		0.46 (0.26–0.80) *p =* 0.006*		*p* = 0.002^*^	
AOR	1.74 (1.00–3.03) *p =* 0.051		0.60 (0.34–1.07) *p =* 0.083			
AOR1	2.02 (1.17–3.48) *p =* 0.011^*^		0.48 (0.27–0.84) *p =* 0.010^*^			
Frequency of dental check-ups (regular vs. irregular)	285/573 (49.7%)	676/1065(63.8%)	193/573 (33.7%)	191/1065(17.9%)	2.62 ± 3.64	1.43 ± 2.74
OR	*0.56 (0.46–0.69)* *p *< 0.001^*^		*2.32 (1.84–2.94)* *p *< 0.001^*^		*p* < 0.001^*^	
AOR	*0.55 (0.44–0.67)* *p *< 0.001^*^		*2.46 (1.94–3.12)* *p *< 0.001^*^			
AOR1	*0.55 (0.44–0.67)* *p *< 0.001^*^		*2.42 (1.91–3.07)* *p *< 0.001^*^			
After 12th month of life before bedtime and at night- water/unsweetened milk only; snacking (sandwiches, fresh fruit); water on a daily basis (vs. unhealthy snacking sporadic and 1–3 times a week)	181/271 (66.8%)	65/149 (43.6%)	50/271 (18.5%)	57/149 (38.3%)	1.23 ± 2.30	3.56 ± 4.38
OR	2.60 (1.72–3.92) *p* < 0.001^*^		0.37 (0.23–0.57) *p* < 0.001^*^		*p* < 0.001^*^	
AOR	2.25 (1.45–3.45) *p* < 0.001^*^		0.47 (0.29–0.75) *p =* 0.002^*^			
AOR2	*2.52 (1.66*–*3.83)* *p* < 0.001^*^		*0.37 (0.24*–*0.59)* *p* < 0.001^*^			
After 12th month of life before bedtime and at night water/unsweetened milk only	733/1171 (62.6%)	231/467(49.5%)	223/1171 (19.0%)	161/467(34.5%)	1.46 ± 2.66	2.82 ± 3.93
OR	*1.71 (1.38*–*2.12)* *p* < 0.001^*^		*0.45 (0.35*–*0.57)* *p* < 0.001^*^		*p* < 0.001^*^	
AOR	*1.59 (1.27*–*1.98)* *p* < 0.001^*^		*0.48 (0.38*–*0.61)* *p* < 0.001^*^			
AOR2	1.68 (1.35–2.080) *p* < 0.001^*^		0.46 (0.36–0.58) *p* < 0.001^*^			
Snacking (sandwiches, fresh fruit)	272/411 (66.2%)	692/1227(56.4%)	83/411 (20.2%)	301/1227(24.5%)	1.49 ± 2.90	1.97 ± 3.21
OR	*1.51 (1.20*–*1.91)* p = 0.001^*^		*0.78 (0.59*–*1.02)* *p = 0.073*		*p* = 0.007^*^	
AOR	*1.44 (1.13*–*1.82)* p = 0.003^*^		*0.84 (0.64*–*1.11)* *p = 0.217*			
AOR2	*1.50 (1.19*–*1.90)* p = 0.001^*^		*0.78 (0.59*–*1.03)* *p = 0.080*			
Drinking water on a daily basis	750/1257 (59.7%)	214/381(56.2%)	281/1257 (22.4%)	103/381(27,0%)	1.69 ± 2.90	2.38 ± 3.76
OR	*1.15 (0.92*–*1.46)* *p = 0.224*		*0.78 (0.60*–*1.01)* *p = 0.059*		*p* < 0.001^*^	
AOR	*1.04 (0.82*–*1.32)* *p = 0.738*		*0.87 (0.67*–*1.14)* *p = 0.318*			
AOR2	*1.12 (0.89*–*1.42)* *p = 0.325*		*0.80 (0.61*–*1.04)* *p = 0.092*			

* *statistical significance p < 0.05*.

a *yes/no concerns protective behaviors presented in the first column*.

b *OR, odds ratio based on simple logistic regression and adjusted OR based on multiple logistic regression; AOR, adjusted for socioeconomic factors; AOR1, adjusted for dietary behaviors-(confounders for inappropriate dietary behaviors, past and present); AOR2, adjusted for hygienic behaviors- confounders for inappropriate hygienic behaviors at present)*.

## Discussion

Protective factors are indicators of preventive activities that may reduce a child's risk of the onset extension of Early Childhood Caries, and should be assessed during the parental interview. Investigating protective factors for ECC and links between diet and tooth brushing in toddlers is essential given the need for early prevention. Many previous studies have examined the risk factors for caries in preschool children. In this study, the focus was on protective factors. To the best of the authors' knowledge, this study is the first one to present protective factors for ECC on a nationally representative sample of 3-year-olds living in all 16 provinces of Poland. One of the important findings of this study is that protective factors for the health of teeth include such dietary behaviors as not snacking or giving any beverages before bedtime and at night, with only water or unsweetened milk given at night, drinking water during the day and healthy snacking (sandwiches and fresh fruit). The right dietary habits are vital in inhibiting development of dental caries (2, 3, 5–10, 12, 13). In this study, when dietary habits in the child's 2nd year of life were analyzed relative to ECC at the age of 3 years, a correlation was observed between consumption of sugar, sweet snacks and sweetened beverages—either its amount, frequency or timing of consumption, addition of sugar to a child's meals. This association is well-known (2, 3, 5–10, 13, 15–21). The correlation between ECC and nursing requires additional debate (11, 12). These study results illustrate the prominent protective role played by healthful dietary practices in preschool children, which is consistent with the results of other authors (2, 3, 20–33). The benefits of water and unsweetened milk, sandwiches and fresh fruit have been proven; this view still holds even after dietary habits conducive to caries have been introduced to the statistic model including the presence of sugar in a child's diet and too frequent consumption of snacks and sweet beverages. The data of the present study are in line with general guidance of providing children with unsweetened milk and water (2, 23, 24, 27, 28, 30, 33). What makes milk and its derivatives cariostatic are its components such as calcium, phosphates, casein, and lipids. There are also many other bioactive ingredients which may also contribute to inhibiting and preventing tooth decay (2, 10, 23). Reduction of caries risk may also be related to the higher frequency of consumption and/or greater amounts (27, 34, 35). The preventive effect of milk on S-ECC was confirmed in Leake et al. (36) and Zaki et al. (27) studies (OR = 0.44; 90% CI = 0.24–0.81 and OR = 0.61; CI = 0.32–1.13, respectively), and supported by the present data. Milk was implicated in reported lower dmfs levels in 3- to 5-year-old African American children (35). In the Johansson et al. (30) study less caries was observed in children who drank milk compared with other beverages (non-sweetened or sweet) consumed with snacks. The above listed studies, as well as the present one, are at variance with the findings of Marshal et al. (37) who reported a neutral association with caries.

Water it is ideal in flushing food debris and reduce the concentration of acids. If water has been fluoridated, it additionally acts topically in the process of remineralization. According to studies by Wang et al. (23, 24), water is the most commonly consumed beverage on a given day among children, most often in Australia (83%) and the US (65%). A similarly high percentage of children in the present study received water as the main beverage (76.8%). Fluoride level in the community water in Poland is below 0.5 mg/L (9). In this study children who drank water had a better chance of being caries-free (OR = 1.15). Quin et al. (38) reported that 75% of caries-free Chinese children would drink only water daily, or mostly water (fluoride concentration in the drinking water did not exceed 0.4 ppm). Özen et al. reported a favorable effect in the reduction of caries if water was drunk following bottle-feeding (OR = 3.45) (39).

Snacking creates a chance to alter diet to include healthy variants: fiber-rich fruit and foods, milk, wholegrain, unrefined cereals, cheese and yogurt that could minimize the action of metabolic acids, and can aid in reversing demineralisation secondary to one's diet (21–28, 31, 32). Healthy diet is always at the forefront of dental prophylaxis since it is assumed that it can neutralize the effect of snacking either by provision of protective ingredients or by replacing bad habits with healthy ones (40). In this study mean dmft index in children with healthy snacking habits was almost three times lower in comparison with children with unhealthy snacking habits. It is fundamental to stress the quality of snacks. The characteristic feature of eating fresh fruit is shorter exposure time in the mouth and the presence of fructose, which has lower cariogenicity than sucrose. The presence of fiber and polyphenols in fruit has been implicated in plaque formation disruption and lowering acidogenicity of oral bacteria (26, 28–31). In the present study, eating fruit between meals reduced the likelihood of decay, similarly to other authors' recommendation that foods rich in NMES should be replaced with fresh fruit and vegetables (26, 27, 30). Bahuguna et al. (32) reported that subjects who consumed a relatively higher amount of fresh fruit were those without caries. Eating five rounds of fruit and vegetables per day is the minimum to maintain dental health among children aged 2–5 years (29). The amount of fruit significantly affected ECC due to its preventive effect [OR = 0.52 (0.26, 1.05)] in Zaki et al. study (27). In the present study, healthy snacking, including fruit, increased significantly the chance of being caries-free, even adjusting for hygienic behaviors (AOR2 = 1.50, *p* = 0.001). Clinical trials have verified how effective fruit are in caries prevention but the results have been inconsistent and inconclusive. Some authors suggest that reasonable fruit intake between meals is not conducive to tooth decay (34). Masson et al. (15) concluded that there was no significant association between whole fruit intake and treatment of decay after adjusting for potential confounding factors. Diurnal reduction of sugar consumption produced an almost 4-fold drop in the incidence of caries (18). In the studied sample 80.8% of parents admitted that they limited sugary foods, however, a detailed analysis did not mean their reduction in the diet. Parents' knowledge and ability to differentiate between healthy and unhealthy foodstuffs was sadly not reflected in what their children actually consumed. Severity of ECC and the need for therapeutic intervention can be dramatically reduced if dietary pattern reflected by the Healthy Eating Index (HEI) and other healthy eating guidelines are adhered to (26–29). Each additional contact with healthy foods resulted in four percent reduction in the caries prevalence rate in Morikawa et al. study (PR = 0.96; 95% CI: 0.92–1.00) (28).

Additionally, when hygiene practices were examined, it was found that each additional daily contact with healthy food was responsible for a drop in the number of carious lesions (28).

Tooth brushing remains a significant protective factor in preventing ECC, which is confirmed by the present study (2, 3, 5). Sujlana and Pannu (41) postulated that brushing twice daily would ensure 1.8 less risk of ECC; the present study also confirmed a significant reduction of ECC when teeth were brushed twice daily (OR = 0.81, *p* = 0.033). Many studies have suggested diet impact on caries, but only few have analyzed whether tooth brushing attenuated the impact of dietary sugars and poor dietary habits, or whether socioeconomic confounders attenuate both. This study addressed a gap in this understanding in preschool children. Another important finding of this study is that brushing a child's teeth twice a day significantly decreases the occurrence of ECC, but this effect was weaker when inappropriate dietary behaviors were also accounted for. This result confirmed findings from previous studies, that tooth brushing does not fully control the impact of diet on dental caries (13, 15). The harmful influence of frequent sugar consumption is not neutralized by tooth brushing. If performed frequently, the latter may decrease the risk related to dental caries, but the children whose diet is sugar-rich will still be likely to develop caries (15). It is the chemical aspect of tooth brushing (fluoride content) which is beneficial, rather than the mechanical one. Along with the study by Masson et al. (15), our participants were given a fluoride-containing toothpaste and the obtained benefit should be attributed more to the presence of fluoride than the frequency of brushing.

In the present study, the chance of being caries-free was higher in children of parents who self-assessed their dentition as healthy. Our finding are supported by other studies, that parents' own oral health associate with dental caries in the primary teeth of their children (5, 21, 34, 42–44).

In the present study, children who admitted to the dentist have a higher prevalence of ECC. A relationship between dental appointments (regular vs. irregular, or none) and the occurrence of caries indicates that the purpose of most visits was the treatment of existing carious lesions and emergency cases and not prophylaxis or check-ups. Similar findings among Egyptian children were reported, where higher caries experience was observed for children who attended dental office (40).

The effect of protective factors was also significant with simultaneous adjustment for confounders (socioeconomic and dietary behaviors) in the present study. Although some studies have reported better oral health associated with higher income (2, 3, 5–7, 10, 16), others have found that children enjoying healthy dentition can be identified in lower socioeconomic background families (22, 45, 46).

The strengths and limitations of the present study must be taken into account. Strengths of this study include a largely homogenous social structure, without different cultures and ethnicities. The large size of the sample of the present study gives it sufficient validity and makes it truly representative of the characteristics of the population. Children were recruited from the general population in the country where caries in preschool children is prevalent, and dental attendance was not relevant in the recruitment process. The choice of one age group of children (3-year-olds only) reduced variation in dental caries levels, allowing better assessment. Children at such young age are more prone to change oral health behaviors as it is still being shaped. The study was carried out by trained and calibrated experienced pediatric dentists to ensure a guarantee of the standardization of their examinations. Standardized survey techniques and methods have provided reliable information. The use of a validated patient chart during clinical examinations instead of reported-caries experience (RCE) and multivariable analysis, are another strengths of the study. The present study evaluated the effect of the consumption of healthy foods in tandem with a cariogenic diet on the prevalence of dental caries among 3-year-old children. All possible variables of feeding practices and oral hygiene behaviors including the impact of potential confounding factors were examined. In spite of that, the study was not devoid of shortcomings like the cross-sectional study design, which prevented determining any causality between ECC and associated factors. Lifestyle behaviors such as the dietary intake could not capture longitudinal changes. Secondly, acknowledged limitation of our study is the self-reported dietary data. Due to the nature of surveys, which are burdened with the risk of errors related to forgetfulness or unwillingness to admit to unhealthy behaviors, that may introduce response bias or social desirability bias. Since information on meals at preschool was not included in the study, the findings referred exclusively to feeding patterns at home. Still, a survey was the preferred method due to the large number of participants and the ease of interpretation—the filling in of the questionnaire was not time-consuming. Yet, questionnaires as a source of information have a low or medium potential, and so obtaining data on the total food intake and oral habits was almost impossible. The points of interest in this study were the presence of foodstuffs with sugar in the diet in the first 2 years of life and snacking, rather than measuring specific dietary contents; however, it was attempted to identify some types of food and beverages recognized as risk factors for ECC. In order to identify protective dietary habits to prevent the occurrence of ECC among young children it will now be necessary to conduct studies examining interactions between specific foods and beverages and how they correlate with dental caries. From this perspective, the results of the present study should be interpreted with caution.

The authors of the present study encountered limitations since radiographs were unavailable, which would potentially lead to higher validation. The same concerns information on non-cavitated lesions. However, the conditions of data collection in preschool environment exclude the use of caries diagnosis of both cavitated and non-cavitated lesions and their current activity status.

In addition, the influence of some residual confounding factors on the findings in the present study cannot be ruled out.

## Conclusions

Preventing a child after 12th month of life from getting any beverages before bedtime, the preference of water or unsweetened milk, sandwiches and fresh fruit as snacks, or water given to a child on a daily basis, tooth brushing twice a day decrease the odds of early childhood caries in 3-year-olds. Diet appears to have primary significance in the etiology of ECC, since tooth brushing can only partly attenuate the impact of inappropriate dietary behaviors on dental caries. Good oral hygiene only minimally decreases the odds of caries in cases of inappropriate dietary behaviors. Children are more often caries-free and have lower severity of caries if their parents' dentition is self-assessed as healthy. These results, in particular, emphasize factors widely regarded as beneficial and protective for the health of teeth. They should guide the practitioners and their patients to better understand these factors.

## Data Availability Statement

The datasets generated for this study can be released on request to the corresponding author following approval of the Ministry of Health.

## Ethics Statement

The research has been conducted in full accordance with ethical principles, including the 1964 Helsinki World Medical Association Declaration of Helsinki and its later amendments. The study protocol was reviewed and ethical clearance was obtained before the study began. The parents of patients/participants provided their written informed consent to participate in this study. The Warsaw Medical University Commission for Bioethics authorised the present study (No. KB/216/2015).

## Author Contributions

DO-K, AT-S, and DG: conception and design of the study, acquisition of data, analysis and interpretation of data, and drafting the article. DO-K: revising it critically for important intellectual content. All authors contributed to the article and approved the submitted version.

## Conflict of Interest

The authors declare that the research was conducted in the absence of any commercial or financial relationships that could be construed as a potential conflict of interest.

## Abbreviations

AAPD: American Academy on Pediatric Dentistry
AOR: adjusted odds ratio
ECC: Early childhood caries
NMES: non-milk extrinsic sugars
OR: odds ratio
S-ECC: severe early childhood caries
WHO: World Health Organization.
